# The utility of DNA extracted from saliva for genome-wide molecular research platforms

**DOI:** 10.1186/s13104-017-3110-y

**Published:** 2018-01-08

**Authors:** Fiona J. Bruinsma, Jihoon E. Joo, Ee Ming Wong, Graham G. Giles, Melissa C. Southey

**Affiliations:** 1 Cancer Epidemiology Centre, Cancer Council Victoria, Melbourne, Australia; 2 Genetic Epidemiology Laboratory, Department of Pathology, University of Melbourne, Parkville, VIC Australia; 3 Precision Medicine, School of Clinical Sciences at Monash Health, Monash University, Clayton, VIC Australia

**Keywords:** Blood, Saliva, Genetic analyses, DNA methylation, Epigenetics

## Abstract

**Objective:**

The study aimed to investigate the suitability of DNA extracted from saliva for high throughput molecular genotyping and DNA methylation platforms by comparing its performance with that of DNA extracted from blood. The genome-wide methylation profile, using the Infinium HumanMethylation450 Beadchip array^®^ (Illumina, San Diego, CA), was measured for 20 DNA samples. Common genetic variation was measured, using the Infinium HumanCore Beadchip^®^ (Illumina, San Diego, CA) for 4 samples (matching samples from 2 people).

**Results:**

DNA from blood and saliva returned genotyping call rates and reproducibility frequencies of > 99%. High-quality DNA methylation data was obtained from both saliva and blood DNA, with average detection p-values for each sample ranging from 0.001 to 0.006. Slightly higher global DNA methylation levels were observed in whole blood DNA than saliva DNA. Correlations between individuals for each sample type were generally greater than correlations between two sample types from the same individual (Pearson’s correlation, r = 0.9696 in 10 pairs of matched blood and saliva derived DNA, r = 0.9702 between saliva samples, and r = 0.9769 between blood derived DNA). Saliva yields DNA of sufficient quantity and quality to compare favourably with blood as a source of DNA for genetic and epigenetic research purposes.

**Electronic supplementary material:**

The online version of this article (10.1186/s13104-017-3110-y) contains supplementary material, which is available to authorized users.

## Introduction

There is increasing interest in both clinical and epidemiological studies in investigating the genetic and epigenetic markers for diseases and their possible interaction with environmental factors. The collection of blood specimens has enabled studies of circulating cells and other blood fractions (e.g. plasma) and supplied considerable quantities of DNA and RNA for analysis. However, this practice is costly, invasive to the research participant, requires trained phlebotomists and laboratory expertise and infrastructure for sample processing and storage.

Many epidemiological studies have begun collecting saliva samples in addition to, or as an alternative to, the collection of blood, as it can be cost-effective and less invasive. Advantages including; (1) samples collected using commercial kits are stable at room temperature and transportable, (2) self-collection kits can be sent to participant’s homes with validated self-guided instructions for providing an adequate sample, and (3) samples can be returned at their convenience. Potential disadvantages of saliva collection include lower mean DNA yield and potential contamination from bacterial DNA [1, 2].

Historically, blood samples have been used as a DNA source for high-density molecular platform analysis, although recently DNA extracted from saliva samples have been successfully used for the detection of germline mutations [3, 4] and for measuring single nucleotide polymorphisms (SNPs) [5]. A challenge is that saliva samples contain multiple enzymes and antibacterial components, as well as large quantities of nucleated buccal (epithelial) cells, leukocytes and bacterial DNA [1, 6, 7], potentially making interpretation more difficult. While there has been variation in reported DNA yield from saliva compared with blood [1, 2] it has been a sufficient template to enable genetic testing and genotype call rates with high concordance [1, 5, 8].

The field of epigenetics has expanded exponentially over the last 15 years. There is increasing interest in the significance of DNA methylation markers to human health. Their potential significance has led to the development of techniques enabling epigenetic markers to be examined across the genome. These methods often rely on the enrichment of methylated DNA using antibodies or methyl-binding substances and most require a large amount of starting DNA [9]. Only one previous study [10] investigated the use of DNA extracted from saliva for methylation analyses.

The Illumina Infinium HumanMethylation450 (HM450K) beadchip array^®^ (San Diego, CA), enables the detection of DNA methylation levels at 485,512 *CpG* dinucleotides across the genome [11]. It requires relatively small amounts of DNA (as low as 500 ng) making it appear feasible for use with DNA extracted from saliva [12].

The aim of the study was to investigate the suitability of DNA extracted from saliva and blood for high-throughput molecular genotyping platforms and whether DNA extracted from saliva samples produced data of the same quality as DNA extracted from a blood sample on the HM450K array and the Illumina Infinium HumanCore array^®^. Generation of methylation measurements from DNA extracted from the two sample types allowed us to examine the extent and the nature of the differences in methylation profiles between DNA extracted from blood and saliva.

## Main text

### Materials and methods

#### Blood and saliva sample collection and DNA isolation

Blood and saliva samples were obtained from a random sample of 10 participants (approximately 0.5% of total participants) enrolled in ongoing studies carried out by the Cancer Council Victoria and collected during a 1 month period. Saliva samples were collected using Oragene^®^ (OG-500) saliva collection kits (DNA Genotek, Ontario, Canada). DNA from saliva was isolated using the salt-out method provided by the manufacturer. DNA was subsequently purified using standard ethanol precipitation, eluted in 800 μl–1 ml 1X TE buffer and stored long-term at 4 °C.

Whole blood samples were collected in a 9 ml EDTA Vacutainer (Becton–Dickinson^®^, Franklin Lakes, New Jersey). DNA was extracted from 2 ml (1 ml × 2) of whole blood using MagNA Pure automated DNA extraction system (Roche^®^, Basel, Switzerland). All DNA samples were quantified using Quant-iT™ Picogreen™ dsDNA assay (Cat No P11496) measured on the Qubit Fluorometer (Life Technologies^®^, Carlsbad, CA) and stored long-term at 4 °C.

#### Bisulfite conversion and the Infinium HM450K

Genomic DNA from blood and saliva (500 ng) was bisulfite converted using EZ DNA Methylation-Gold^®^ kit (Cat No D5006) (Zymo Research, Irvine, CA), as per the manufacturer’s instruction. 200 ng of bisulfite converted DNA was whole-genome amplified overnight and fragmented. The DNA was precipitated and resuspended in a hybridisation buffer and hybridised onto the HM450K Beadchip overnight. The single-base extension and staining steps were performing using the Freedom EVO^®^ automated liquid handler (TECAN, Männedorf, Switzerland).

#### Illumina HumanCore-12^®^ Beadchip

Common genetic variation was measured for 4 samples (matching samples from 2 individuals). Genomic DNA from blood and saliva (500 ng)were provided to the Australian Genome Research Facility (Melbourne, Australia) and the Illumina Infinium HumanCore-12^®^ Beadchip assay run as per manufacturer’s instructions.

#### Data processing

Raw intensity signals from iScan were exported into R environment (R programming software v3.0.3). The data was processed using the *minfi* R package available from Bioconductor [13]. The data was normalised using the subset-quantile within array normalization (SWAN) to reduce potential technical bias from the platform’s two types of probes [9]. Probes with detection p-values > 0.05 were considered as background noise and subsequently removed. As no sex-specific analysis was anticipated, probes on X and Y chromosomes were also removed. β-values and M-values from a total of 471,899 probes were calculated in the *minfi* using the formulae: *β* = *Meth/(Meth* + *Unmeth* + *100)* and M = *log* (*Meth/Unmeth*).

Raw data from the HumanCore-12 Beadchip was imported into the GenomeStudio v2011.1 Genotyping module 1.9.4 software (Illumina, San Diego, CA) and processed using the software default settings. The Humancore-12v1-0_a manifest and cluster files were used for data quality assessment and analysis as per manufacturer’s instructions.

### Results

#### DNA isolated from blood and saliva

We successfully isolated genomic DNA from both saliva and blood samples from all 10 study participants. There were 7 males and 3 females aged between 51 and 70 years old at the time of collection. Four had a diagnosis of prostate cancer and six a diagnosis of kidney cancer. A total mean DNA yield of 64.1 μg (range 3.9–176.0 μg) was obtained from 3.3 ml of saliva, giving a mean yield per ml of 18.5 μg/ml (range 1.2–44.0 μg). A mean of 8.5 μg (range 3.2–22.6) of DNA was obtained from 2 ml of whole blood, with a mean yield per ml of 4.3 μg/ml (1.6–11.3 μg).

#### Measurement of genetic variation

Based on matching samples from 2 individuals from the Illumina Infinium HumanCore-12^®^ array, both blood and saliva samples returned high quality data with SNP call rates and reproducibility frequencies of > 99%.

#### DNA methylation data obtained from saliva DNA

**Fig. 1:**
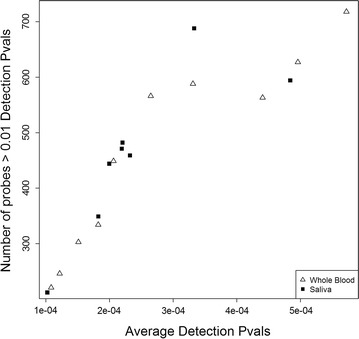
Plot of average detection p-values and number of probes with > 0.01 detection p-values for individual samples

High quality genome-wide DNA methylation data was obtained from matching saliva and blood DNA using the HM450K array. Average detection p-values across all 485,512 probes for each sample ranged from 0.0001 to 0.0006, and no individual sample had more than 806 probes with detection p-values > 0.01 (Fig. 1). There was no noticeable difference in data quality between saliva and blood samples. We observed slightly higher global DNA methylation levels in DNA from whole blood samples (average β-value 0.4963, 95% CI 0.4899–0.5028) than DNA from saliva (average β-value 0.4879, 95% CI 0.4832–0.4928), when using average β-values across all detected probes (471,899) as surrogate measurements.

**Fig. 2:**
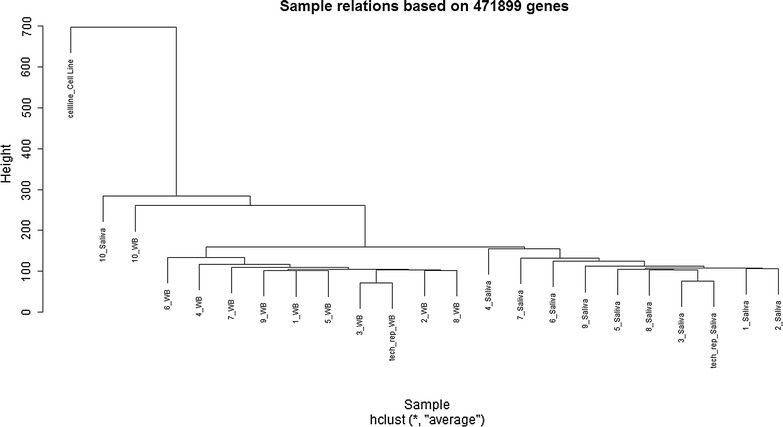
Hierarchical clustering model of all saliva and whole blood samples based on all detected probes (471,899)

In order to compare DNA methylation similarities between the two sample types and between individuals, we performed a multidimensional scaling analysis (based on all detected probes). Samples tended to cluster by sample type rather than individuals (Fig. 2). Methylation of DNA from whole blood samples was more uniform between individuals than were the DNA from saliva samples. Correlations between DNA of the same sample type were generally greater than correlations between DNA of different sample types (from the same individuals) (Pearson’s correlation, *r* = 0.9696 in 10 pairs of blood and saliva samples and *r* = 0.9702 between all saliva samples, and *r* = 0.9769 between all blood samples) (Additional file 1: Table S1).

#### Tissue-specific DNA methylation marks

DNA methylation marks of saliva and whole blood samples were found to be highly source specific and we identified a large set of consistently differentially methylated probes between the two source types. An F-test performed on our 10 paired samples found that approximately a quarter of all detected probes (127,860) were significantly differentially methylated (FDR adjusted *p* value < 0.05) (Additional file 2: Table S2).

#### Correlative methylation marks between saliva and whole blood derived DNA

**Fig. 3:**
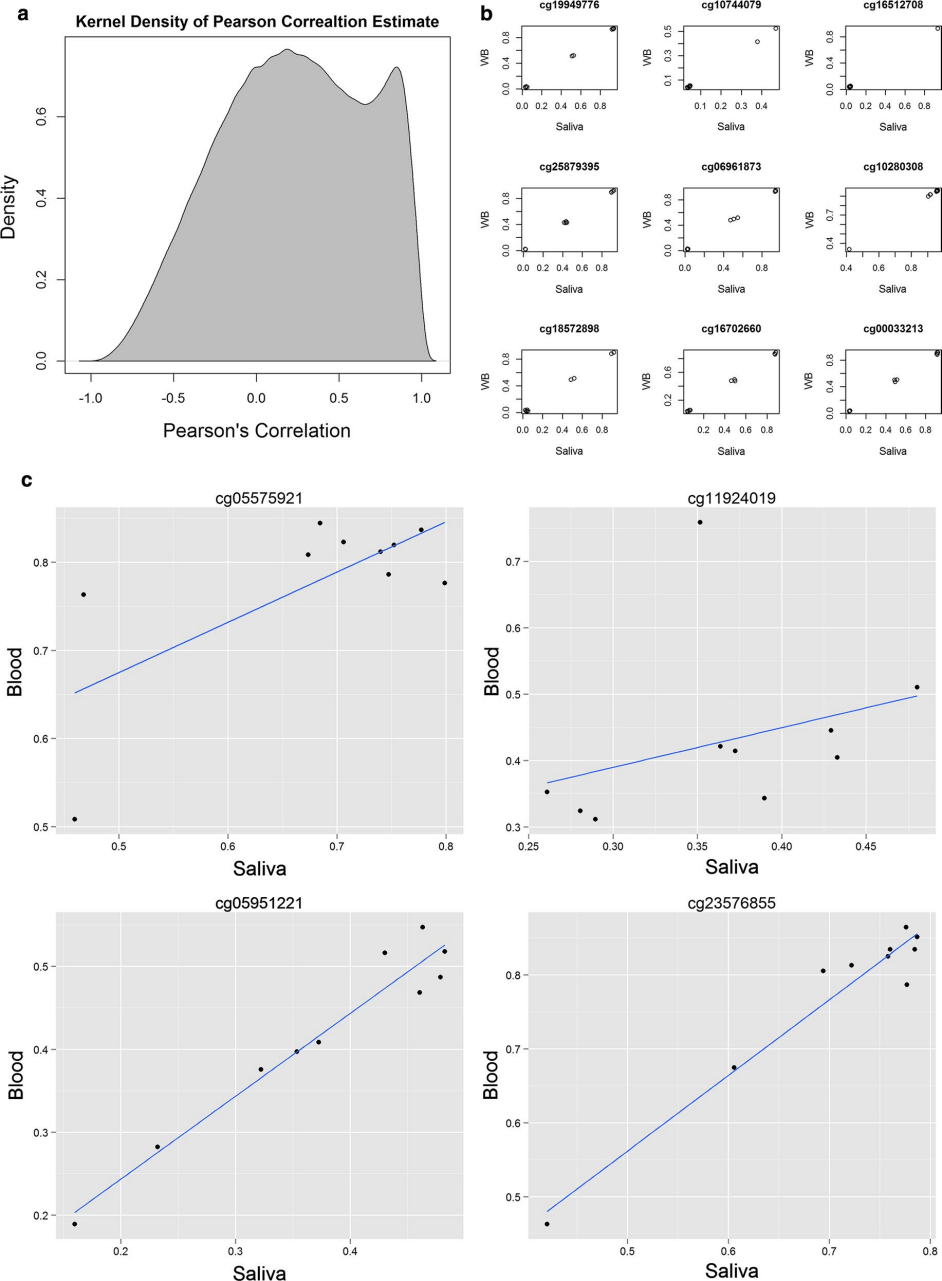
**a** Density plot showing pair-wise Pearson’s correlation between DNA derived from saliva and blood samples from 10 participants. **b** Scatter plots of two source types on the top 9 most correlative probes. **c** Scatter plot showing correlation between two source DNA types on probes cg05575921, cg05951221, cg11924019, cg23576855

To identify correlative methylation marks between paired DNA sources, we calculated Pearson’s paired rank correlation for each of 471,899 probes. There were a large proportion of positively correlated probes between two sources (Fig. 3a). We found 68,870 probes showing moderate to strong correlation between two source types (p-value < 0.01, r > 0.7646) (Additional file 3: Table S3). Only 2712 of these probes were negatively correlated. In order to investigate whether these correlations were biological or a technical artefact of the platform, we checked for overlapping SNPs within these correlative probes. According to the Illumina SNPs annotation table (v3), a large proportion (25,443) of these probes overlapped known SNPs. Given most SNPs are not source-specific, unlike DNA methylation marks, the majority of these correlative methylation marks may have arisen due to the technical limitation (i.e. overlapping SNPs within probes). To investigate this further, the top 9 most correlative probes were plotted (Fig. 3b) and a strong grouping of these samples into 3 groups was observed, suggesting that these methylation signals may actually be driven by underlying genetic polymorphisms. Care should be taken in interpreting DNA methylation results from this platform.

Wu et al. compared a number of methylation markers that are correlative between blood and saliva in young female individuals and found moderate correlation in some markers [4]. We tested four of these markers (cg05575921, cg05951221, cg11924019, cg23576855) on our dataset and we found strong correlations for two probes (cg059512221, r = 0.9722, 95% CI 0.8830–0.9936; cg23576855, r = 0.9728, 95% CI 0.8858–0.9938; Fig. 3c) (Additional file 4: Table S4).

### Discussion

Collecting saliva samples is a non-invasive and convenient method to obtain biological specimens from study participants. The results of our study show that we were able to obtain a higher quantity of DNA from saliva than whole blood samples of the same volume and is consistent with findings reported by Hansen et al. [14]. Current literature [1, 6, 8] and our broader experience suggests that DNA yields from saliva samples can be quite variable for several reasons including variation in pre-collection mouth content, washing and the DNA extraction method. The use of saliva DNA for a variety of genomic analyses has been previously demonstrated [3–5] and we were able to replicate high call rates on high density SNP arrays consistent with findings from other studies [1, 4, 15].

The HM450K array data quality matrix of each sample was high and did not differentiate between DNA source. We found that DNA methylation marks were much more similar within each DNA source (Fig. 2). We found almost a quarter of the 471,899 probes were significantly differentially methylated between the two sources, consistent with DNA methylation tissue-specificity [10]. Overall, saliva DNA methylation was slightly more variable than blood derived DNA (Fig. 2 and Additional file 1: Table S1). This is most likely due to the variability in cell composition of saliva samples (they are likely to include a proportion of epithelial and haematological cell lineages). A further study of comparing saliva cell count between samples may be beneficial. However, we believe that DNA obtained from saliva samples is a viable alternative to that derived from blood samples for methylation analyses.

### Limitations

A large set of correlated methylation marks across the source of DNA (within individuals) were identified. Some of these DNA methylation marks may have been identified due to cell type similarities (e.g. leucocytes) or biologically uniform methylation marks between two sources of DNA. The HM450K DNA methylation detection technique is somewhat limited when SNPs overlap probes which can be misinterpreted as DNA methylation changes. As genetic polymorphisms are uniform across all tissues within individuals a proportion of the correlation between DNAs are due to this technical limitation.

## Additional files

**Additional file 1: Table S1.** Pearson’s correlation coefficient matrix between individual samples.

**Additional file 2: Table S2.** List of differentially methylated probes (FDR adjusted p-value < 0.05) between saliva and whole blood samples.

**Additional file 3: Table S3.** List of probes showing moderate to strong within-individual correlation between saliva and whole blood samples.

**Additional file 4: Table S4.** Pearson’s correlation coefficient between blood and saliva of probes cg05575921, cg05951221, cg11924019, cg23576855.

## Abbreviations

SNP: single nucleotide polymorphism
HM450K: Illumina Infinium HumanMethylation450
SWAN: subset-quantile within array normalization
